# A Cross Sectional Study of Knowledge and Attitudes Towards Tuberculosis amongst Front-Line Tuberculosis Personnel in High Burden Areas of Lima, Peru

**DOI:** 10.1371/journal.pone.0075698

**Published:** 2013-09-19

**Authors:** Mark Minnery, Carmen Contreras, Rosa Pérez, Ninfa Solórzano, Karen Tintaya, Judith Jimenez, Silvia Soto, Leonid Lecca

**Affiliations:** 1 University of Queensland, School of Population Health, Brisbane, Australia; 2 Socios en Salud Surcusal Peru, Lima, Peru; 3 Dirección de Salud V Lima Ciudad; Ministerio de Salud. Lima, Peru; McGill University, Canada

## Abstract

**Introduction:**

Tuberculosis, reported as the second most common infectious cause of death worldwide, is a key mortality contributor in developing countries and globally. The disease is endemic in Peru and while relative success was achieved during the 1990s in its control, this slowed as new complications, such as multi drug resistant TB arose. Health centre workers participating in the national DOTS program, create the front-line TB work-force in Peru meaning their knowledge and attitudes about the disease are key in its control.

**Methods:**

A Spanish language, multiple choice knowledge and attitudes survey was designed based on previous successful studies and the national Peruvian TB control guidelines. It was applied to two health networks in Lima, Peru amongst 301health workers participating in the national TB control program from 66 different health centres. The study results were analysed to test mean knowledge scores amongst different groups, overall gaps in key areas of TB treatment and control knowledge, and attitudes towards the disease and the national TB control program.

**Results:**

A mean knowledge score of 10.1 (+/- 1.7) out of 15 or 67.3% correct was shown. Demographics shown to have an effect on knowledge score were age and level of education. Major knowledge gaps were noted primarily in themes relating to treatment and diagnostics. Greater community involvement including better patient education about TB was seen as important in implementing the national TB control program. Participants were in disagreement about the current distribution of health resources throughout the study area.

Discussion Serious knowledge gaps were identified from the survey; these reflect findings from a previous study in Lima and other studies from TB endemic areas throughout the world. Understanding these gaps and observations made by front-line TB workers in Lima may help to improve the national TB control program and other control efforts globally.

## Introduction

In 2009 tuberculosis (TB) was reported as the second most common infectious cause of death worldwide. In the same year approximately 9.2 million new cases of TB and 1.7 million associated deaths occurred, primarily in the developing world [1,2]. Control of the disease has been highlighted as a priority for the World Health Organization (WHO) and as such is a key component of the Millennium Development Goal 6.

A clear example of the concerning state of TB control is Peru, which in 2011 recorded 15% of all reported TB cases in the region of the Americas. Additionally it was the country with the largest total number of reported cases of Mulit-Drug Resistant Tuberculosis (MDR-TB) in the region [3]. With an incidence rate of 109.7 per 100,000 (2011), each year Peru has more than 30,000 new cases of TB [4]. Within Peru, the concentration of the disease is highest in the country’s capital, Lima. Here approximately 58% of the country’s TB cases, 82% of MDR-TB cases and 93% of the country’s Extremely Drug Resistant (XDR-TB) cases reside [4,5].

Directly Observed Therapy (DOT) has now become the accepted standard for TB treatment throughout the world, and has been applied in Peru with relative success since the 1990s [6]. A standard DOT program will involve the administration of TB antibiotics to patients under the direct observation of health program staff. Unfortunately with the appearance of MDR-TB the fight against TB in Peru has become more difficult and the reduction in reported cases apparent in the nineties has slowed [6,7].

Building on the DOT programs, governing bodies have agreed that the most effective procedures for reducing prevalence, drug-resistance and treatment abandonment for TB involve opportune diagnosis reinforced with an efficient system of reporting, follow-up and new case identification [7–9]. With this in mind it is important to note that the effective performance of patient-managed care programs are often reliant on the number, distribution, knowledge, skills and levels of motivation and competence of supporting health personnel [10].

Additionally the WHO has estimated that, of the five main obstacles to the expansion of DOT and the successful development of local and national programs, four are directly human resource related [9]. As such if human resources are scarce or undevdeveloped the diagnosis and appropriate management of patients with TB and their contacts will be weakened.

For this reason, it is necessary to conduct assessments of the knowledge and attitudes about TB amongst front-line health personnel in charge of TB care, in order to identify potential problems, limitations and areas for improvement. Front-line TB health personnel in this context can be defined as health personnel who form the primary contact point amongst the community for accessing the national TB control program. They may include medical doctors, nurses, nurse technicians, community health workers (health promoters) and other allied health personnel. For this study we broadened this term to include laboratory staff, given their integral role in primary TB services.

Front-line TB personnel in Peru receive mixed forms and levels of training depending on their position within the health system. Doctors must complete at lesat 6 years of higher education, a 1-3 year period of residence/specialisation and a year of rural service, nurses and biologists (lab staff) must study for 4 years along with the completion of a thesis and additionally nurses must complete a year as an intern and a year of rural service in order to practice in the national health program. Technical staff including nursing and laboratory staff usually complete at least 3 years of study often from private institutions of varied quality and 6 months internship in a hospital. Health promoters are community members working voluntarily or semi-voluntarily, who are trained for a period of at least 2 weeks in various primary care and promotional activities. The role of the health promoter involves the provision of primary care services throughout the community and often forms the first contact point between the community and access to the public health system.

We conducted a comprehensive survey of firstly, knowledge of front-line TB health personnel working in the Peruvian National TB control program in order to ascertain knowledge gaps in key areas relating to tuberculosis control and prevention; and secondly, of front-line TB personnel attitudes towards current themes facing the Peruvian national TB control program. It was hoped that with greater understanding of the gaps which face these two areas of TB control it may be possible to contribute to strengthening the National TB control program and, of global significance, provide insights into the issues which face front-line TB personnel in other high TB and MDR-TB burdened areas.

## Methods

A Spanish language, multiple choice survey (available as Information S1) was created based on previous studies [11–16] and the national guidelines for the diagnostics and treatment of TB [17]. Each survey contained 48 questions divided into three sections: participant demographics, tuberculosis knowledge, and attitudes about tuberculosis and corresponding issues facing Peru. Demographics detailed important considerations about the participant which were thought to have a role in knowledge of and attitudes towards TB such as age, gender, reported profession, TB specific training and disease status of participants and people they live with. Professions were divided into medical doctor, nurse, nurse technician, health promoter and ‘others’ (which encompassed all other front-line TB workers such as laboratory staff). The knowledge section contained 15 questions and was again divided into three sections: epidemiology and transmission of TB (5 questions), diagnostics of TB (3 questions), and treatment of TB (7 questions). Answers were established as either correct or incorrect, but participant’s indicating more than one choice for a question or if a question was left unanswered were marked as incorrect. The survey was first piloted then revised in August 2011 to front-line TB nurses in non-study areas of Lima.

The attitudes section contained 21 questions covering topics relating to the quality and level of education of health personnel in the national tuberculosis control program, the level of education relating to tuberculosis and its treatment in the community, access and barriers to access for treatment, resources devoted to the national tuberculosis control program, treatment adherence and local tuberculosis control program priorities. Attitudes were recorded using a 5-point Likert scale.

The study was applied in the two health networks in Lima, Peru (San Martin de Porres-Rimac-Los Olivos and Lima) in areas of high TB incidence, including 16 districts of high incidence representing 37% of the population of Lima [4]. From these two networks 301 health personnel from 66 government health centres participated. Health centres cover a wide range of services including primary care, preventative services and diagnostic testing and are often the first point of contact for suspected tuberculosis patients within the national control program. The health centres also play an important role in TB education and prevention activities amongst the community.

The survey was conducted from September 2011-February 2012. Only health care personnel who at the time were working in the national tuberculosis control program were considered for the study. The national program was selected as large proportion of the TB response in Lima is provided by public institutions. As an example it was reported that in 2010 approximately 56% of sympotomatic patients sought national TB control program establishments for diagnostics. Additionally 76% of TB diagnosis occurred in national control program establishments [18].

Ethics approval was given by the human subjects committees of the National Institute of Health in Lima, Peru and all participants were first given a detailed explanation of the questionnaire and its motives and were then guaranteed anonymity and informed that participation was entirely voluntary. Following verbal consent the survey was administered on the same day wherever possible.

### Analysis

All surveys were collated into Excel for analysis. For each participant’s responses to the knowledge section, four scores were applied. An overall knowledge score (0-15), a transmission of TB score (0-5), diagnostics of TB (0-3), and treatment of TB (0-7). All questions were given a binary score, either correct or incorrect. In the instance where more than one answer had been selected the participant was given a score of 0 for that question; equally if a participant failed to answer a question a 0 was applied. The differences in mean knowledge were quantified for profession, age, gender, TB course participation, ever having had TB and having someone living in the same household with TB.

A 5-point Likert scale was used in attitude questions indicating degree of agreement. Questions related to topics such as attitudes to individuals with TB and opinion of the current status of the Peruvian health system in response to TB.

Mean knowledge scores were compared amongst groups using a two-sample t test. Linear regression was then used to compare variables associated with mean knowledge score. Attitude questions were collapsed into agreement, neutral or disagreement. Fischers exact test was used to determine agreement on attitude questions.

## Results

### Knowledge

As can be seen in Table 1, demographics varied amongst study participants. The vast majority were female (77.41%) and working as doctors (15.82%), nurses (21.88%) or nurse technicians (22.22%). The proportion of participants with more than one year working with the national program within their current health centre was 83%, working within the health centre’s TB program was 69% and working in TB generally was 72%.

**Table 1:** Participant Demographics.

**Participant Demographics**		N=301
	N	%
**Participant Sex**		
M	68	22.59%
F	233	77.41%
**Participant Age**		
20-25	12	3.99%
26-30	23	7.64%
31-35	31	10.30%
36-40	52	17.28%
41-45	57	18.94%
46-50	51	16.94%
51-55	45	14.95%
56-60	15	4.98%
60+	15	4.98%
**Participant Profession**		
Doctor	48	15.82%
Nurse	65	21.88%
Nurse Technician	67	22.22%
Health Promoter	43	13.80%
Other	78	26.26%
**Participated in TB training in the last 12 months**		
Yes	139	46.18%
No	161	53.49%
**Participants who have been diagnosed with active TB**		
Yes	11	3.65%
No	290	96.35%
**Participants who live with someone they know to have had active TB**		
Yes	56	18.60%
No	245	81.40%

As indicated in Table 2 mean knowledge score was 10.1 (+/- 1.7) out of a possible 15 or 67.3% correct. Age had an effect on mean knowledge score which decreased from the 20-25 age group at 11.81 (+/- 2.3) to the 55-60 at 9.37 (+/- 1.8) (P <0.01). This excluded the 65+ age group who scored relatively highly; however, whose results should be taken with caution given the relatively small sample size. Mean knowledge score was shown not to be significantly different in relation to gender, having had training related to TB in the last 12 months or having had TB and/or having lived with someone who had TB. When comparing amongst professions, those associated with a higher level of education had a greater average knowledge score but this decreased with shorter required education time. Doctors had the highest mean score at 10.4 (+/- 1.4) to health promoters who had 9.5 (+/-1.8) (p < 0.01). The ‘others’ group received a mean score of 9.9 (+/- 1.8).

**Table 2:** Knowledge Scores total and by profession.

**Number and % correct response among profession towards all knowledge questions**						
**Question**	**Total (N=301**)	**Medical Doctor (N=47**)	**Nurse (N=65**)	**Nurse Technician (N=67**)	**Health Promoter (N=43**)	**Other (N=78**)
What is the germ which causes tuberculosis	298 (99.0)	46 (97.9)	65 (100)	65 (97.0)	40 (93.0)	78 (100)
Is TB a transmittable disease?	288 (95.7)	43 (91.5)	61 (93.8)	64 (95.5)	40 (93.0)	76 (97.4)
How is TB transmitted?	291 (96.7)	45 (95.7)	65 (100)	63 (94.0)	39 (90.7)	75 (96.1)
Who has higher risk in developing the TB disease?	177 (58.8)	33 (70.2)	41 (63)	42 (62.7)	18 (41.9)	42 (53.8)
Do all people with the TB infection develop the TB disease?	163 (54.2)	39 (83.0)	42 (64.6)	23 (34.3)	14 (32.6)	45 (57.6)
What is the most common symptom of pulmonary (TB of the lungs) TB?	228 (75.7)	36 (76.6)	54 (83)	42 (62.7)	26 (60.5)	67 (85.8)
Which is the most effective tool in the diagnosis of pulmonary TB?	73 (24.3)	10 (21.3)	13 (20)	23 (34.3)	9 (20.9)	15 (19.2)
How many sputum samples are necessary for collection to diagnose TB?	253 (84.1)	35 (74.5)	60 (92.3)	58 (86.6)	33 (76.7)	67 (85.8)
Is it possible to cure TB?	294 (97.7)	46 (97.9)	63 (96.9)	65 (97.0)	41 (95.3)	75 (96.1)
How long (in months) is a complete treatment for pulmonary TB under the primary scheme?	286 (95.0)	46 (97.9)	64 (98.4)	64 (95.5)	40 (93.0)	72 (92.3)
How many drugs are used in the primary scheme for treatment of pulmonary TB?	263 (87.4)	45 (95.7)	63 (96.9)	62 (92.5)	32 (74.4)	61 (78.2)
What is Mult Drug Resistant TB (MDR-TB)?	112 (37.2)	19 (40.4)	25 (38.4)	27 (40.3)	17 (39.5)	24 (30.7)
What is the frequency for follow up sputum tests under the primary scheme for pulmonary TB?	51 (16.9)	3 (6.4)	15 (23)	17 (25.4)	3 (7.0)	9 (11.5)
What is the most effective form for evaluating the results of a complete treatment scheme?	107 (35.5)	14 (29.8)	21 (32.3)	30 (44.8)	16 (37.2)	25 (32)
What are the consequences of an incomplete or abandoned treatment?	147 (48.8)	29 (61.7)	29 (44.6)	24 (35.8)	20 (46.5)	43 (55.1)
Mean score	10.1 +/- 1.7	10.4 +/- 1.4	10.4 +/- 1.5	10.1 +/- 1.7	9.5 +/- 1.8	9.9 +/- 1.8

Amongst knowledge sub-themes by and large the worst knowledge scores were seen in the treatment section with a total participant average of 4.2 (+/- 1.1) out of 7 or 60% correct. Of equal concern was the average diagnostic score of 1.89 (+/- .67) out of 3 or 61% correct. With a small amount of variance 4.09 (+/- 0.77) out of 5 or 81% correct was achieved for the epidemiology and transmission section.

Knowledge gaps pertaining to treatment were shown in relation to consequences of inadequate treatment and MDR-TB and the frequency at which patient follow up procedures should be administered. Only 50.39% of participants were able correctly identify all consequences of an inadequate treatment regime; additionally only 42.17% were able to correctly describe what constitutes MDR-TB. Just 20.45% participants knew the correct frequency at which follow up sputum tests were required following the national TB control guidelines. Only 35.65% of participants were able to identify the ideal method for evaluating the results of a complete treatment regime.

Other significant gaps were noted such as only 61.45% of participants being able to identify all persons at higher risk of developing active pulmonary TB once infected. In line with other studies a low percentage (55.38% of participants) were able to identify that not all people with the TB infection developed symptoms.

### Attitudes

As can be seen in table 3 several themes gained strong agreement amongst participants. A majority (99.2%) of participants agreed that more community involvement was needed to improve TB control and prevention and also that educating patients about TB was an important part of the TB treatment regime. Continuing along this theme there was a near consensus (89.4%) that there were often difficulties in helping patients to understand why they needed to complete treatment after they had started feeling better. Themes which showed mixed attitudes were primarily surrounding logistics and health system support. For example, only roughly half of participants (54.8%) felt that the laboratory services which covered their health centre were inadequate for dealing with the load of tests required to effectively treat patients at their health centre. Participants were also in disagreement (53.8% agreement) as to whether their health centre had sufficient personnel to treat all TB participants who arrive at their centre for treatment.

**Table 3:** Profession vs attitudes.

			**The use of traditional or alternative treatments against TB makes the current TB situation in Peru worse**		**The staff in your health establishment seek help from their peers or superiors for managing difficult TB cases**		**The lack of adequate knowledge amongst the community about TB makes seeking treatment for TB more difficult for TB patients**		**The current methods used to treat TB are accepted by your TB patients**		**The majority of staff working at your health establishment have had adequate training to perform their given tasks**		**This health establishment contains an adequate system for monitoring TB patients during treatment**		**The laboratory installations which your health establishment uses are adequate for your needs**		**Your health establishment has sufficient personnel to treat all the TB patients which come to your health establishment**		**The Knowledge and awareness of TB in your community is adequate**		**The majority of your community know about the services which your health establishment offers for the diagnosis and treatment of TB**		**The supply system for TB medication in your health establishment is adequate**
**Overall**	**Agreement**		81.7%		94.8%		89.1%		85.6%		74.9%		81.4%		54.8%		53.8%		43.7%		64.6%		90.4%
	**Neutral**		7.5%		3.0%		5.5%		5.3%		8.5%		5.4%		8.5%		5.4%		18.3%		13.1%		3.0%
	**Disagreement**		10.5%		2.2%		4.8%		8.8%		15.5%		13.1%		36.4%		40.5%		37.7%		21.8%		6.6%
**Doctor**	**Agreement**		93.9%		95.5%		87.9%		92.4%		72.7%		87.9%		57.6%		50.0%		39.4%		63.6%		89.4%
	**Neutral**		3.0%		3.0%		7.6%		1.5%		9.1%		4.5%		10.6%		7.6%		10.6%		15.2%		4.5%
	**Disagreement**		3.0%		1.5%		4.5%		4.5%		15.2%		7.6%		30.3%		42.4%		48.5%		21.2%		6.1%
**Nurse**	**Agreement**		84.1%		98.6%		92.8%		89.9%		72.5%		87.0%		53.6%		55.1%		40.6%		72.5%		95.7%
	**Neutral**		2.9%		1.4%		0.0%		4.3%		8.7%		7.2%		8.7%		2.9%		20.3%		7.2%		1.4%
	**Disagreement**		13.0%		0.0%		5.8%		5.8%		18.8%		5.8%		37.7%		42.0%		39.1%		18.8%		2.9%
**Nurse Technician**	**Agreement**		75.4%		92.8%		89.9%		91.3%		65.2%		79.7%		58.0%		55.1%		47.8%		62.3%		92.8%
	**Neutral**		10.1%		2.9%		4.3%		4.3%		15.9%		4.3%		13.0%		7.2%		21.7%		14.5%		1.4%
	**Disagreement**		13.0%		4.3%		4.3%		4.3%		18.8%		15.9%		29.0%		37.7%		30.4%		23.2%		5.8%
**Health Promoter**	**Agreement**		73.7%		97.4%		86.8%		76.3%		89.5%		76.3%		65.8%		57.9%		50.0%		68.4%		89.5%
	**Neutral**		13.2%		2.6%		10.5%		7.9%		5.3%		2.6%		5.3%		2.6%		23.7%		13.2%		2.6%
	**Disagreement**		13.2%		0.0%		2.6%		15.8%		2.6%		21.1%		28.9%		39.5%		26.3%		18.4%		7.9%
**Other**	**Agreement**		81.4%		89.8%		88.1%		78.0%		74.6%		76.3%		39.0%		50.8%		40.7%		55.9%		84.7%
	**Neutral**		8.5%		5.1%		5.1%		8.5%		3.4%		8.5%		5.1%		6.8%		15.3%		15.3%		5.1%
	**Disagreement**		10.2%		5.1%		6.8%		13.6%		22.0%		15.3%		55.9%		40.7%		44.1%		27.1%		10.2%
				**Finding all of the new case of TB is an important task in controlling the disease**		**It is important to realise more actions to include the community in TB prevention and control**		**For patients with TB it is difficult to understand the need to continue taking medication after they have started feeling better**		**There is large difference in treatment completion if a TB treatment is administered through direct observation (DOT**)		**Educating patients about TB is an important part of their treatment**		**Drug resistant TB is an important problem in Peru**		**TB patients in Peru are confronted with a significant social stigma surrounding the disease**		**Health financial resources would be better spent educating the public about TB than directly into the observation and treatment of TB**		**The manner in which TB patients receive their medication needs to be adaptable and take into account the individual circumstances of the patient**		**Your current health post has clear guides for diagnosis and treatment of TB**	
**Overall**		**Agreement**		97.7%		99.2%		89.4%		95.6%		100.0%		99.4%		94.7%		78.2%		82.0%		93.8%	
		**Neutral**		0.0%		0.0%		3.0%		1.5%		0.0%		0.0%		2.3%		4.7%		3.5%		3.5%	
		**Disagreement**		2.3%		0.0%		7.6%		2.3%		0.0%		0.6%		2.7%		15.0%		13.2%		2.4%	
**Doctor**		**Agreement**		98.5%		98.5%		92.4%		98.5%		100.0%		100.0%		98.5%		75.8%		75.8%		95.5%	
		**Neutral**		0.0%		0.0%		0.0%		1.5%		0.0%		0.0%		1.5%		3.0%		3.0%		1.5%	
		**Disagreement**		1.5%		0.0%		7.6%		0.0%		0.0%		0.0%		0.0%		18.2%		19.7%		3.0%	
**Nurse**		**Agreement**		97.1%		100.0%		94.2%		97.1%		100.0%		98.6%		100.0%		69.6%		79.7%		94.2%	
		**Neutral**		0.0%		0.0%		1.4%		1.4%		0.0%		0.0%		0.0%		7.2%		4.3%		4.3%	
		**Disagreement**		2.9%		0.0%		4.3%		0.0%		0.0%		1.4%		0.0%		21.7%		14.5%		0.0%	
**Nurse Technician**		**Agreement**		95.7%		100.0%		81.2%		87.0%		100.0%		100.0%		89.9%		76.8%		79.7%		94.2%	
		**Neutral**		0.0%		0.0%		5.8%		4.3%		0.0%		0.0%		1.4%		7.2%		4.3%		2.9%	
		**Disagreement**		4.3%		0.0%		13.0%		7.2%		0.0%		0.0%		7.2%		11.6%		13.0%		2.9%	
**Health Promoter**		**Agreement**		97.4%		97.4%		89.5%		97.4%		100.0%		100.0%		92.1%		84.2%		86.8%		92.1%	
		**Neutral**		0.0%		0.0%		2.6%		0.0%		0.0%		0.0%		5.3%		2.6%		2.6%		5.3%	
		**Disagreement**		2.6%		0.0%		7.9%		2.6%		0.0%		0.0%		2.6%		13.2%		10.5%		2.6%	
**Other**		**Agreement**		100.0%		100.0%		89.8%		98.3%		100.0%		98.3%		93.2%		84.7%		88.1%		93.2%	
		**Neutral**		0.0%		0.0%		5.1%		0.0%		0.0%		0.0%		3.4%		3.4%		3.4%		3.4%	
		**Disagreement**		0.0%		0.0%		5.1%		1.7%		0.0%		1.7%		3.4%		10.2%		8.5%		3.4%	

Several differences were seen in terms of attitude scores amongst different employment groups. Agreement that patients accept current TB treatment methods gained a near complete agreement amongst doctors (92.4%) as opposed to only three quarters of health promoters. While in general approximately half of health personnel agreed or were neutral that laboratory installations at their respective health establishments were adequate, the others group, of which a large proportion is laboratory personnel, tended more towards disagreement.

## Discussion

In agreement with a previous study [16], it has been shown that serious gaps exist in the knowledge of front-line TB personnel about TB in highly endemic areas of Lima, Peru. This mirrors findings from other developing countries facing similar TB burdens. Two surveys surrounding the diagnosis and treatment of TB in urban areas of Kenya showed that doctors were often not aware of the correct diagnosis and treatment procedures for dealing with TB patients [19,20]. Although in a rural setting, Vietnamese health care providers showed a low (67%) knowledge score using a similarly structured survey [14]. Comparisons such as these show that low TB knowledge scores among health personnel are not isolated to Peru but endemic in other high burden areas. The importance of this is outlined below.

As has been shown elsewhere [16] the consequences of inadequate TB treatment are of serious concern for the Peruvian national TB control program. Given the nature of MDR-TB it is reasonable to assume that under or mis-education about the disease can result in case complications and this in turn may lead to drug resistance [21]. As such the low knowledge rates among participants in questions relating to both inadequate treatment outcomes and MDR-TB classification should be highlighted as a key area for intervention.

Questions relating to the frequency of follow up sputum tests may show that knowledge of national TB standards and guidelines is poor. This should be considered an area for future improvement amongst front-line TB personnel and the national TB program as a whole. Equally, low knowledge of TB risk factors should be further investigated. Increasingly it is being shown that TB burdens are significantly heightened by a variety of risk factors as the disease is frequently accompanied by HIV, diabetes and malnutrition [22]. As such further steps to strengthen education surrounding identification and prevention of accompanying risk factors may contribute to the national TB control program in Peru and elsewhere.

In line with the findings of Kiefer et al [16] a significant proportion of front-line TB personnel were unaware that not all people infected with the TB bacterium develop symptoms. This misconception combined with the gap in knowledge relating to TB risk factors creates an area of concern given the integral role which front-line TB personnel play in relating information about the disease throughout the community.

The views taken by the front-line TB personnel in relation to TB are highly pertinent both to Peru and the global fight against TB. As it is front-line personnel who gain firsthand experience confronting the disease, often on a daily basis, they may be best able to identify unique bottlenecks to improved TB control. As such the consensus found in this study relating to the need for greater community involvement may be taken into account when reviewing disease control programs both in Lima and abroad. The perceived lack of understanding amongst TB patients relating to the non-completion of treatment may show a need for greater community involvement in national program design. This sentiment is reinforced by the positive response from study participants about the need for community involvement in the national TB program. Equally the discord amongst personnel relating to access to both sufficient laboratory services and personnel to cover the needs of patients may indicate an inequitable distribution of TB resources in the National TB program. The ‘others’ group, of which a large proportion was made up of laboratory staff, agreed more strongly on the inadequacy of laboratory services. These people may have unique insights into the current situation of laboratory services indicating a greater need than is perceived by the rest of the TB workforce. Health promoters’ lower perception that patients were accepting of current treatment methods could indicate a more sensitized view of the current sentiment within the community. As health promoters’ primary responsibility is community integration they may be able to more accurately demonstrate the current opinion of the community. These remain areas for further research.

Two limitations to this study were noted. Firstly the cross-sectional design and limited study area meant that the knowledge and attitudes of personnel may not be representative of those at the national level. Taking this into account the number of TB personnel covered within the study region are highly representative as a proportion of the total front-line TB personnel in Lima. Additionally similar findings from other studies in both rural and urban sectors add weight to the results [16,19–21]. Secondly, while all attempts were made to provide the best possible environment in which to undertake this survey, Peruvian TB personnel are both critically short of time and extremely dedicated to their patients, both factors that leave little time for surveys, so in other more controlled environments knowledge and attitude responses may have slightly differed.

This study confirms findings from similar studies in both Lima, Peru, and similarly TB affected countries. While further investigation is needed into the nuances surrounding front-line TB worker knowledge and factors that affect it, it is clear that gaps exist and that efforts must be made in order to reinforce and support health personnel. Additionally consensus and disagreement in attitudes amongst front-line TB personnel present important areas of further research which may have the potential to highlight unique problems systemic in National TB control programs. There are problems which front-line TB personnel may be best able to identify, and addressing these may increase the effectiveness and quality of global TB control efforts both in Peru and throughout the world.

## Supporting Information

Information S1 **Survey Instrument.**
(DOCX)
