# Supplementary material for: A Cross Sectional Study of Knowledge and Attitudes Towards Tuberculosis amongst Front-Line Tuberculosis Personnel in High Burden Areas of Lima, Peru
Source: PLoS One. 2013 Sep 19;8(9):e75698. doi: 10.1371/journal.pone.0075698 (PMC3777965; doi:10.1371/journal.pone.0075698)
Supplement: Information S1 — Survey Instrument. (DOCX) [file pone.0075698.s001.docx]

Anexo 1:
**Encuesta sobre Tuberculosis**

**Características de los participantes:**

1. Nombre del establecimiento de salud al que pertenece: ________________________
2. Cargo que tiene en el establecimiento de salud: ______________________________
3. Edad: _____ años
4. Sexo:
5. Masculino
6. Femenino
7. Nivel de formación:
8. Médico.
9. Enfermero(a).
10. Técnico de enfermería.
11. Enfermero serumista.
12. Promotor de salud.
13. Otro (Especificar): _____________________________________
14. Nivel educativo (*Sólo para ser respondido por los Promotores de salud*):
15. Superior universitario completo.
16. Superior universitario incompleto.
17. Superior técnico completo.
18. Superior técnico incompleto.
19. Secundario universitario completo.
20. Secundario universitario incompleto.
21. Otro (Especificar): _____________________________________
22. ¿Cuánto tiempo labora o trabaja en este establecimiento de salud?
23. 0 a menos de 3 meses.
24. De 3 meses a menos de 6 meses.
25. De 6 meses a menos de 12 meses.
26. De 1 año a más.
27. ¿Cuánto tiempo labora o trabaja en el programa de TB del establecimiento de salud?
28. 0 a menos de 3 meses.
29. De 3 meses a menos de 6 meses.
30. De 6 meses a menos de 12 meses.
31. De 1 año a más.
32. ¿Cuánto tiempo labora o trabaja en actividades relacionadas a la TB, incluso si este trabajo se realiza(ó) fuera del establecimiento de salud?
33. 0 a menos de 3 meses.
34. De 3 meses a menos de 6 meses.
35. De 6 meses a menos de 12 meses.
36. De 1 año a más.
37. ¿En los últimos 12 meses ha participado en algún entrenamiento/curso sobre Tuberculosis?
38. Sí (Especificar cantidad): ____ curso(s) y ______ horas de entrenamiento.
39. No
40. ¿Ha estado enfermo en algún momento de Tuberculosis?
41. Sí
42. No
43. ¿Ha tenido o tiene a alguien cercano a Ud. (por ejemplo: familiar o amigo) enfermo de Tuberculosis?
44. Sí
45. No

**Preguntas sobre Conocimiento de Tuberculosis:**

***Epidemiología y transmisión de la TB:***

1. Cuál es el germen que produce la TB?
2. *Mycobacterium tuberculosis.*
3. *Mycobacterium avium.*
4. *Mycobacterium pneumoniae.*
5. *Mycobacterium contagiosum.*
6. La TB es una enfermedad transmisible?
7. Sí.
8. No.
9. No sé.
10. Cómo se transmite la TB?
11. Vía aérea.
12. Por contacto físico.
13. Vía sexual.
14. Todas las anteriores.
15. No sé.
16. Quiénes tienen riesgo alto de desarrollar TB? (Marque TODAS las opciones posibles)
17. Personas con VIH.
18. Contactos cercanos de personas con TB.
19. Diabéticos.
20. Personas con enfermedades crónicas.
21. Embarazadas.
22. Todas las personas con infección TB desarrollarán enfermedad TB (tendrán síntomas)?
23. Sí.
24. No.
25. No sé.

***Diagnóstico de TB:***

1. Cuál es el síntoma más común de TB Pulmonar?
2. Fiebre.
3. Tos.
4. Estornudos.
5. Cansancio.
6. Dolor de pecho.
7. Cuál es la herramienta de diagnóstico más efectiva para TB Pulmonar?
8. Examen de los microorganismos de una muestra de esputo.
9. Test de PPD.
10. Radiografía de tórax.
11. Cultivo de muestra sanguínea.
12. Cuántas muestras de esputo son necesarias colectar para diagnóstico?
13. 1.
14. 2.
15. 3.
16. 4.

***Tratamiento de TB:***

1. La TB puede curarse?
2. Sí.
3. No.
4. No sé.
5. Cuánto dura (en meses) el tratamiento de un paciente con TB pulmonar que recibe un esquema primario?
6. 2 meses.
7. 6 meses.
8. 9 meses.
9. 12 meses.
10. Cuántos medicamentos se emplean en el tratamiento de un paciente con TB pulmonar que recibe un esquema primario?
11. 2.
12. 3.
13. 4.
14. 5.
15. Qué es la tuberculosis multidrogo-resistente o TB-MDR?
16. Es cuando el bacilo es resistente a todos los medicamentos que existen actualmente para tratar la TB.
17. Es cuando el bacilo es resistente al menos a la isoniacida y a la pirazinamida.
18. Es cuanro el bacilo es muy agresivo y se necesita al menos de 8 a 12 meses de tratamiento.
19. Ninguna de las anteriores.
20. Con qué frecuencia se conduce el seguimiento de esputo en los pacientes con TB en tratamiento?
21. Monitoreo de esputo mensual.
22. Monitoreo de esputo quincenal.
23. Monitoreo de esputo trimestral.
24. Monitoreo de esputo anual.
25. Cuál es la mejor forma para evaluar los resultados del tratamiento?
26. Radiografía de tórax.
27. Test de PPD.
28. Examen de esputo.
29. Todas las anteriores.
30. No sé.
31. Cuáles son las consecuencias de un tratamiento inadecuado/incompleto?
    1. TB resistente.
    2. Contagio a otras personas.
    3. Desarrollo de TB extrapulmonar/sistémica.
    4. Todas las anteriores.
    5. No sé.

**Preguntas sobre Actitudes respecto a la Tuberculosis:**

1. Encontrar todos los casos nuevos de TB es un desafío importante para el control de la TB:
   1. Totalmente de acuerdo.
   2. De acuerdo.
   3. Indiferente.
   4. En desacuerdo.
   5. Totalmente en desacuerdo.
2. Es importante realizar más acciones para involucrar a la comunidad en el control y prevención de la tuberculosis:
   1. Totalmente de acuerdo.
   2. De acuerdo.
   3. Indiferente.
   4. En desacuerdo.
   5. Totalmente en desacuerdo.
3. A los pacientes con TB a menudo les resulta difícil entender por qué tienen que seguir tomando pastillas después que empiezan a sentirse mejor:
   1. Totalmente de acuerdo.
   2. De acuerdo.
   3. Indiferente.
   4. En desacuerdo.
   5. Totalmente en desacuerdo.
4. Hay una gran diferencia en el cumplimiento del tratamiento por el paciente si esta se administra bajo terapia de observación directa (DOT):
   1. Totalmente de acuerdo.
   2. De acuerdo.
   3. Indiferente.
   4. En desacuerdo.
   5. Totalmente en desacuerdo.
5. Educar a los pacientes acerca de la tuberculosis es una parte importante de su tratamiento:
   1. Totalmente de acuerdo.
   2. De acuerdo.
   3. Indiferente.
   4. En desacuerdo.
   5. Totalmente en desacuerdo.
6. La tuberculosis resistente es un problema importante en el Perú:
   1. Totalmente de acuerdo.
   2. De acuerdo.
   3. Indiferente.
   4. En desacuerdo.
   5. Totalmente en desacuerdo.
7. Los pacientes con TB en el Perú a menudo se enfrentan a un significativo estigma social y a la vergüenza:
   1. Totalmente de acuerdo.
   2. De acuerdo.
   3. Indiferente.
   4. En desacuerdo.
   5. Totalmente en desacuerdo.
8. El dinero sería mejor gastado en educar a la gente en el Perú acerca de la tuberculosis que en la observación de tratamiento de la tuberculosis:
   1. Totalmente de acuerdo.
   2. De acuerdo.
   3. Indiferente.
   4. En desacuerdo.
   5. Totalmente en desacuerdo.
9. La forma en que los pacientes reciben sus pastillas para la TB debería ser adaptable y tomar en cuenta las circunstancias individuales de cada paciente:
   1. Totalmente de acuerdo.
   2. De acuerdo.
   3. Indiferente.
   4. En desacuerdo.
   5. Totalmente en desacuerdo.
10. Su establecimiento de salud tiene guías claras para el diagnostico y tratamiento de la TB:
    1. Totalmente de acuerdo.
    2. De acuerdo.
    3. Indiferente.
    4. En desacuerdo.
    5. Totalmente en desacuerdo.
11. El uso de tratamientos tradicionales o alternativos de la TB hace que la situación de esta enfermedad en el país sea peor, pues complica el tratamiento:
    1. Totalmente de acuerdo.
    2. De acuerdo.
    3. Indiferente.
    4. En desacuerdo.
    5. Totalmente en desacuerdo.
12. El personal de su establecimiento de salud busca ayuda en sus niveles superiores para el manejo de casos difíciles:
    1. Totalmente de acuerdo.
    2. De acuerdo.
    3. Indiferente.
    4. En desacuerdo.
    5. Totalmente en desacuerdo.
13. La falta de conocimiento adecuado de la comunidad sobre la TB hace que sea difícil para los pacientes buscar tratamiento para la TB:
    1. Totalmente de acuerdo.
    2. De acuerdo.
    3. Indiferente.
    4. En desacuerdo.
    5. Totalmente en desacuerdo.
14. Los métodos de tratamiento de la TB que utilizamos son aceptados por nuestros pacientes:
    1. Totalmente de acuerdo.
    2. De acuerdo.
    3. Indiferente.
    4. En desacuerdo.
    5. Totalmente en desacuerdo.
15. La mayoría del personal de su establecimiento de salud ha tenido o tiene un entrenamiento adecuado para las actividades que realiza:
    1. Totalmente de acuerdo.
    2. De acuerdo.
    3. Indiferente.
    4. En desacuerdo.
    5. Totalmente en desacuerdo.
16. Este establecimiento de salud cuenta con un sistema eficaz para monitorear cercanamente a los pacientes con TB durante su tratamiento:
    1. Totalmente de acuerdo.
    2. De acuerdo.
    3. Indiferente.
    4. En desacuerdo.
    5. Totalmente en desacuerdo.
17. Las instalaciones del laboratorio que utilizan en el establecimiento de salud para diagnosticar TB son adecuadas para sus necesidades:
    1. Totalmente de acuerdo.
    2. De acuerdo.
    3. Indiferente.
    4. En desacuerdo.
    5. Totalmente en desacuerdo.
18. Tienen suficiente personal para tratar todos los pacientes con TB que vienen a su establecimiento de salud:
    1. Totalmente de acuerdo.
    2. De acuerdo.
    3. Indiferente.
    4. En desacuerdo.
    5. Totalmente en desacuerdo.
19. Los conocimientos y la toma de conciencia sobre la TB en su comunidad es adecuada:
    1. Totalmente de acuerdo.
    2. De acuerdo.
    3. Indiferente.
    4. En desacuerdo.
    5. Totalmente en desacuerdo.
20. La mayoría de personas de su comunidad conoce sobre la disponibilidad de los servicios con que cuentan su establecimiento de salud para el diagnóstico y tratamiento de la TB:
    1. Totalmente de acuerdo.
    2. De acuerdo.
    3. Indiferente.
    4. En desacuerdo.
    5. Totalmente en desacuerdo.
21. El sistema de suministro de medicamentos para TB de su establecimiento de salud es adecuado:
    1. Totalmente de acuerdo.
    2. De acuerdo.
    3. Indiferente.
    4. En desacuerdo.
    5. Totalmente en desacuerdo.
